# Occurrence of Staphylococcal Ocular Infections of Food Producing Animals in Nsukka Southeast, Nigeria

**DOI:** 10.1155/2014/528084

**Published:** 2014-02-12

**Authors:** Sunday Ositadinma Udegbunam, Rita Ijeoma Udegbunam, Madubuike Umunna Anyanwu

**Affiliations:** ^1^Department of Veterinary Surgery, University of Nigeria, Nsukka 400001, Nigeria; ^2^Department of Veterinary Pathology and Microbiology, Faculty of Veterinary Medicine, University of Nigeria, Nsukka 400001, Nigeria

## Abstract

Staphylococcal ocular infections of food animals have been somewhat under diagnosed probably due to the ubiquitous nature of staphylococcal organisms. This study was undertaken to determine the occurrence of staphylococcal ocular infections of food producing animals in Nsukka Southeast, Nigeria, and to determine the antibiogram of the isolated staphylococci. A total of 5,635 food producing animals were externally examined for signs of clinical ocular conditions. Animals that showed clinical eye lesions were further examined using pen light to assess the entire globe and the pupillary reflex. Blindness was assessed using menace blink reflex, palpebral reflex and obstacle methods. Isolation and identification of staphylococcal isolates from ocular swabs were done by standard methods. Antibiogram of the isolates was determined by disc diffusion method. Sixty-three (1.1%) of the examined animals showed signs of ocular condition. Thirty-one (49.2%) of the cultured swabs yielded *Staphylococcus aureus* (*S. aureus*). Isolation rates from different animal species were caprine (60%), ovine (33.3%), bovine (12.5%), and porcine (0%). Resistance of the isolates was 100% to ampicillin/cloxacillin, 90% to tetracycline, 80% to streptomycin, 71% to chloramphenicol, 20% to erythromycin, 16% to gentamicin, and 0% to ciprofloxacin and norfloxacin. Twenty-five (81%) of the isolates were multi-drug resistant. This study has shown that antibiotic-resistant staphylococci are associated with a sizeable percentage of ocular infections of food producing animals and should be considered during diagnosis and treatment.

## 1. Introduction

Although the eyes are maintained in “near sterile state” by the eyelids and biological secretions such as precorneal tear film, lysozyme, lactoferrin, secretory immunoglobulins, and defensins, a breakdown of the systemic and/or local defense mechanism allows colonization by opportunistic pathogenic bacteria in the air, dust, skin, and formite and on vectors, to which the eyes are constantly exposed [1–3]. This colonization by bacterial organisms results in ocular infections which generally manifest clinically as blepharospasm, excessive lacrimation, mucopurulent discharges, keratitis, conjunctivitis, corneal oedema, corneal ulceration, corneal opacity, descemetocele, possible ocular rupture, and loss of vision [4–7]. These clinical signs are not pathognomonic to a specific microbial ocular infection and hence occur in most bacterial ocular infections. Ocular infections may affect one eye (unilateral) or both eyes (bilateral) in mild and acute infection and/or severe and chronic infections [8].

In food producing animals, ocular infections affect crucial behaviours such as appreciating the environment, locating the food, negotiating objects in familiar territories, and interacting with flock mates [9]. This consequently affects the fertility, fecundity, weight gain, milk yield, and market value of infected animals [8, 10, 11]. However, reports from different countries have shown that various bacterial organisms such as *Moraxella* spp., *Mycoplasma conjunctivae*, *Branhamella ovis*, *Chlamydia* spp., *Rickettsia* spp., *Listeria monocytogenes*, *Corynebacterium pyogenes*, *Staphylococcus aureus*, *Staphylococcus intermedius*, *Escherichia coli*, *Bacillus* spp., *Streptococcus pyogenes*, *Arcanobacterium pyogenes*, *Proteus* spp., *Pasteurella haemolytica,* and *Pseudomonas aeruginosa* are associated with ocular infections of food animals [4, 5, 8, 12–14]. Amongst these organisms, *Staphylococcus* species have been incriminated either as the sole cause or as concurrent pathogen in different ocular infections of food producing animals [4, 5, 7, 8, 13]. But in most ocular infections of food producing animals, *Staphylococcus* has been somewhat underdiagnosed probably due to its ubiquitous nature and nonspecific clinical characteristics shown by the animals during infection. Moreover, with the rise in isolation of antibiotic-resistant *Staphylococcus* in animals, treatment of *Staphylococcus*-associated ocular infections is becoming increasingly difficult. This consequently results in increase in cost of food animal production [8, 11].

In Nigeria, treatment of infected animals without conducting sensitivity test is a common practice by nonveterinarians and farmers. In staphylococcal ocular infections, this indiscriminate practice may result in selection pressure and development of multiple resistance to many antibiotics by the associated organism. These organisms can transfer resistance genes to other microorganisms in the animal. Because there are no strict regulations guiding animal slaughter in Nigeria, these infected animals are often slaughtered, thereby posing health risks to the consumers. Therefore, studies on resistance profile of *Staphylococcus* isolates from ocular infections of food animals will provide basis for empiric treatment of these infections.

However, there is no information on the occurrence and/or antibiogram of *Staphylococcus* organisms associated with ocular infections of food animals (cattle, goats, sheep, and pigs) in Nigeria. The only reported study on bacterial ocular infection of food animal in Nigeria which was in form of a case report was that of Ojo et al. [5]. In this study, *Moraxella bovis* was associated with an outbreak of keratoconjunctivitis in a goat herd in Southwest, Nigeria. The objectives of this study were to determine the occurrence of *Staphylococcus*-associated ocular infections of food producing animals in Nsukka, Southeast Nigeria, and to determine the antibacterial resistance profile of the staphylococcal isolates.

## 2. Materials and Methods

### 2.1. Study Location and Sampling of Animals

This cross-sectional study was carried out from February 2008 to February 2012. Seven towns (Opi, Obollo-Eke, Ogbede, Orba, Adada, Ihealumona, and Nsukka municipal centers) in Nsukka were selected purposively for this study. Each town was visited once to avoid the possibility of resampling. Following adequate restraint, the eyes of 5,635 animals (cattle, goats, sheep, and pigs) kept in farms, households, and sales barns were externally examined under day time light. Animals that showed signs of ocular condition were subsequently further examined using penlight to evaluate the entire eye globe and to assess the pupillary light reflex. They were also assessed for loss of vision using menace blink reflex, palpebral reflex, and obstacle test methods [8, 15]. The species of each affected animal and the clinical sign(s) shown by it were recorded accordingly.

### 2.2. Isolation and Identification of Staphylococcus Species

After disinfection of eyes with clinical lesions, ocular swabs were taken using sterile swab sticks moistened with sterile normal saline. Precaution was taken to ensure that swabs did not touch the eyelid skin by opening the animal's eye wide and rotating swab stick forth and back on the corneal surface and conjunctiva. Swab samples were transported aseptically in ice packs within 2 hours of collection to the Microbiology Laboratory of the Department of Veterinary Pathology and Microbiology, University of Nigeria, Nsukka. The samples were inoculated into nutrient broth (Oxoid) and incubated aerobically at 37°C for 24 hours. For selective isolation of *Staphylococcus*, a loopful of each broth culture was inoculated onto mannitol salt agar and incubated at 37°C for 18–24 hours. Colonies were further subcultured on 5% sheep blood agar and incubated at 37°C for 24 hours. Identification of the *Staphylococcus* isolates was based on colonial characteristics, microscopic characteristics, and biochemical characteristics such as production of haemolysis, catalase, urease, deoxyribonuclease, clumping factor, free coagulase and oxidase, and fermentation of sugars according to standard protocols [16]. The identified isolates were maintained on nutrient agar slope at 4°C until needed.

### 2.3. Determination of Antibacterial Profile of Staphylococcal Isolates

This was carried out following the disc diffusion procedure [17]. The staphylococcal isolates were sub-cultured on nutrient agar, incubated at 37°C for 24 hours. Then colonies for each isolate were adjusted to 0.5 McFarland's turbidity standard in sterile phosphate buffered saline. The standardized broth culture was used to cover the entire surface of sterile Mueller-Hinton agar plates using sterile swab sticks.

Eight antibiotic discs (Oxoid) consisting of five antibiotic classes were used. They include gentamicin (10 **μ**g), streptomycin (10 **μ**g), erythromycin (5 **μ**g), ciprofloxacin (5 **μ**g), norfloxacin (5 **μ**g), tetracycline (30 **μ**g), ampicillin/cloxacillin (10 **μ**g/10 **μ**g), and chloramphenicol (30 **μ**g). The discs were placed strategically on each inoculated Mueller-Hinton agar plate and incubated at 37°C for 18 hours. After incubation, the zone of inhibition around each disc was measured with a meter rule. Each test was performed in triplicate and the mean inhibitory zone diameter (IZD) was calculated for each isolate and each antibiotic to the nearest whole millimetres. The mean IZD was interpreted as susceptible, intermediate, or resistant according to the Clinical and Laboratory Standards Institute (CLSI) [18] criteria.

### 2.4. Statistical Analysis

Data generated were analysed descriptively and expressed in percentages.

## 3. Results

### 3.1. Occurrence of Clinical Signs of Ocular Condition in Food Producing Animals

Out of the 5635 animals examined, 63 (1.1%) consisting of 47 (2.1%) goats, 8 (0.3%) cattle, 6 (1.1%) sheep, and 2 (0.4%) pigs showed various signs of ocular condition (Table 1).

### 3.2. Isolation Rates of *S. aureus* from Ocular Swabs of Food Producing Animals

Of the 63 swab samples cultured, 31 (49.2%) yielded *S*. *aureus*. Isolation rate from different animal species was as follows: caprine (60%), bovine (12.5%), ovine (33.3%), and porcine (0%) (Table 2). Of the 31 isolates obtained, 17 (55%) was obtained from animals showing mucopurulent discharges, 9 (29%) from those with keratitis, 4 (13%) from those with conjunctivitis, and 1 (3%) from those with *conjunctiva nictitans* prolapse (Table 3).

### 3.3. Antibiogram of *S. aureus* Isolates from Food Producing Animals

The *S*. *aureus* isolates showed highest resistance to ampicillin/cloxacillin (100%), followed by tetracycline (90%), streptomycin (80%), chloramphenicol (71%), erythromycin (20%), and gentamicin (16%). None (0%) of the isolates was resistant to ciprofloxacin and norfloxacin (Table 4). Twenty-five (81%) of the isolates showed multidrug resistance to 3 or more of the antibiotic classes tested (Table 5).

## 4. Discussion

The occurrence of 1.1% clinical ocular conditions observed in this study suggests that ocular conditions may not be common clinical findings in food animals reared in Nsukka Southeast, Nigeria. This may not be unconnected to the fact that most of these animals are reared intensively and therefore they are less prone to ocular infections. However, the highest species occurrence (2.1%) of clinical ocular condition observed in goats may be related to the fact that most goats reared in Nsukka are of the West African Dwarf (WAD) goat breeds. This breed of goat is reared extensively on free grazing and scavenging system [19] with little or no veterinary care. These goats roam about and frequently fight while competing for food and flock mates. This behavior predisposes them to ocular infections. The least species occurrence (0.4%) of clinical ocular condition in pigs may be related to the fact that pigs are raised intensively in Nsukka; hence, they were less prone to ocular infections.

The mucopurulent discharges observed in 21 (0.4%) of the total examined animals suggest that there was infection by pyogenic organism(s). This was supported by the fact that 17 (55%) *S*.* aureus* were isolated from animals that had eye clinical lesions. However, other pyogenic organisms such as *Streptococcus* could also have contributed to the suppuration. The ocular discharges may have occurred due to secondary *S*. *aureus* infection consequent upon breakdown in local defenses of the eyes following systemic infection and/or immune depression [20]. Isolation of 9 (29%) *S*.* aureus* from animals with keratitis suggests that the organism may be the cause of the lesion. Inflammatory reaction by the phagocytic cells may have caused the keratitis. Egwu et al. [21] reported isolation of *S*. *aureus *from ovine keratitis. Prolapse of *conjunctiva nictitans* observed in 1 (2.6%) of the affected animals may suggest that ocular conditions requiring surgical intervention are not common in food producing animals reared or sold in Nsukka area. Nevertheless, isolation of 1 (3%) *S*. *aureus* from the eyes of the animal may suggest that secondary bacterial ocular infection may have occurred following prolapse.

The isolation rate (49.2%) of *S*. *aureus* (Table 2) obtained in this study incriminated this organism as a potential cause of ocular infections of food animals in Nsukka Southeast, Nigeria. In Southeast Ethiopia, Takele and Zerihun [13] reported 15% *S*. *aureus* isolation from cattle with keratoconjunctivitis. In Norway, Åkerstedt and Hofshagen [4] reported 5% *S*. *aureus* isolation rate from sheep with keratoconjunctivitis. In India, Rajesh et al. [7] isolated 83.3% *S*. *aureus* from buffaloes with keratoconjunctivitis, whereas, Abdullah et al. [8] isolated *S. aureus* from a Friesian cow with stage III keratoconjunctivitis in Malaysia. *S*. *aureus* has been reported to elicit virulent factors (adhesins) which enables it to colonize ocular tissues [20].

The high rate (80%) of resistance by the *S*. *aureus* isolates to streptomycin and tetracycline may be as a result of frequent and indiscriminate use of these drugs by nonveterinarians and farmers in treatment of food animals in Nsukka. In treating these animals, streptomycin is often combined with penicillin to exert a broad spectrum activity. This may have resulted in selection pressure and resistance among the isolates. High rate (100%) of resistance to ampicillin/cloxacillin shows that the isolates exerted complete selection pressure and resistance to these beta-lactam antibiotics. This high resistance may have developed because these drugs are commonly used in preparation of animal drugs and they have been tremendously abused by indiscriminate use by farmers in Southeast Nigeria [22]. This high resistance to the beta-lactams could have been mediated by production of beta-lactamase—the commonest mechanism of beta-lactam resistance in *Staphylococcus* [23]. The *Staphylococcus* isolates could also have acquired antibiotics resistance genes from other organisms in their environment, being a normal flora in the environment, skin, and mucous membranes of animals [6, 8].

Furthermore, high rate (71%) of chloramphenicol resistance suggests that the isolates were exposed to the drug. Chloramphenicol is a component of most antibiotic eye drops used in human and veterinary practice, and being available over the counter, it is possible that some of the farmers used it in an attempt to treat the infected animals. This led to the development of resistance by the *Staphylococcus* isolates. This high rate of chloramphenicol resistance calls for concern about the use of this drug in food animals in Nigeria. It suggests that, despite the international ban on the use of chloramphenicol in food producing animals (due to its carcinogenic effect) [24], some farmers in Nsukka still use it in treating their animals. It has been long documented that opthalmological use of chloramphenicol also causes blood dyscrasia [25]. Thus, this finding in food animal isolates in Nsukka poses great health risk to consumers.

Moreover, the low resistance rates to gentamicin (5%) and erythromycin (20%) suggest that the isolates have not developed complete resistance to the drugs. Low rate (0%) resistance of the isolates to fluoroquinolones (ciprofloxacin and norfloxacin) indicates that the isolates were highly susceptible to this class of antibiotics. This high rate (100%) of susceptibility may not be unconnected to the fact that fluoroquinolones are not commonly used in treating food animals in Nsukka area, except for poultry. Also erythromycin and gentamicin (banned because of its nephrotoxic effects) [26] are not aminoglycosides of choice in food producing animal therapy.

The fact that 25 (81%) of the isolates were resistant to 3 or more of the antibiotic classes tested suggests that the isolates have acquired multidrug resistance genes. It also suggests that the isolates could have been exposed to different antibiotics thereby exerting selection pressure against them. This is significant in empirical treatment of ocular infections because, without conducting antibiotic sensitivity test, treatment of these infections would be difficult.

In conclusion, this study has shown that *Staphylococcus *is associated with ocular infections of food producing animals in Nsukka Southeast, Nigeria. The occurrence of ocular conditions in food animals in the study area during the period of this study is 1.1%, while the prevalence of *S*. *aureus*-associated ocular infection is 49.2%. Therefore, though staphylococcal eye infections are rarely diagnosed in food producing animals because of its ubiquitous nature, *S*. *aureus* may have to be considered more commonly as a causative agent of eye infections in food producing animals reared and/or sold in Nsukka Southeast, Nigeria.

Since the *S*. *aureus* isolates appeared to be more resistant to the commonly used antibiotics in the study area, it is recommended that ocular infections in food animals should be treated with veterinary preparations which are chosen carefully after the antibiogram of the organism is known. From this study, the best drugs for the treatment of staphylococcal ocular infections of food animals are the fluoroquinolones—ciprofloxacin and norfloxacin. Because streptomycin, tetracycline, and ampicillin/cloxacillin acid have been seriously compromised therapeutically, they are probably of no current value in the treatment of staphylococcal ocular infections in Nsukka area, and therefore their use should be curtailed.

## Figures and Tables

**Table 1:** Occurrence of clinical signs of ocular condition in food producing animal species.

Clinical signs	Number of animals with clinical signs				Total (%)
	Bovine (*n* = 2316)	Caprine (*n* = 2285)	Ovine (*n* = 534)	Porcine (*n* = 500)	
Mucopurulent discharge	2	16	2	1	21 (0.4)
keratitis	2	6	1	0	9 (0.2)
Bilateral conjunctivitis	4	24	3	1	32 (0.6)
Prolapse of *conjunctiva nictitans *	0	1	0	0	1 (0)

Total (%)	8 (0.3)	47 (2.1)	6 (1.1)	2 (0.4)	63

%: percentage.

**Table 2:** Isolation rate of *S*. *aureus* from different food producing animal species.

Animal species	Number of cultured samples	Number of *S*. *aureus *isolated	Percent of *S*. *aureus* isolated
Bovine	8	1	12.5
Caprine	47	28	60
Ovine	6	2	33.3
Porcine	2	0	0

Total	63	31	

**Table 3:** Isolation rate of *S*. *aureus *from food producing animals with specific clinical signs.

Clinical sign	Number (%) of *S*. *aureus* isolated
Mucopurulent discharge	17 (55)
Keratitis	9 (29)
Conjunctivitis	4 (13)
Prolapse of *conjunctiva nictitans *	1 (3)

Total	31 (100)

%: percentage.

**Table 4:** Antibiogram of *Staphylococcus aureus* isolated from food producing animals.

Antibiotics	Number (%) of *Staphylococcus aureus* strains (*n* = 31)		
	Resistant	Intermediate	Susceptible
Gentamicin	5 (16)	6 (19)	20 (65)
Streptomycin	25 (81)	6 (19)	0 (0)
Erythromycin	6 (20)	25 (80)	0 (0)
Ciprofloxacin	0 (0)	0 (0)	31 (100)
Norfloxacin	0 (0)	0 (0)	31 (100)
Tetracycline	25 (80)	6 (20)	0 (0)
Ampicillin/cloxacillin	31 (100)	0 (0)	0 (0)
Chloramphenicol	22 (71)	0 (0)	9 (29)

%: percentage.

**Table 5:** Number of antibiotic classes to which isolates were resistant.

Number of antibiotic classes	Number (%) of isolates resistant
1	6 (19)
2	0 (0)
3	3 (10)
4	22 (71)

Total	31 (100)

%: percentage.
